# Noninvasive Evaluation of Intraventricular Flow Dynamics by the HyperDoppler Technique: First Application to Normal Subjects, Athletes, and Patients with Heart Failure

**DOI:** 10.3390/jcm11082216

**Published:** 2022-04-15

**Authors:** Andrea Fiorencis, Marco Pepe, Vittorio Smarrazzo, Marika Martini, Salvatore Severino, Valeria Pergola, Marco Evangelista, Pierluigi Incarnato, Marco Previtero, Marco Maglione, Sabino Iliceto, Gianni Pedrizzetti, Donato Mele

**Affiliations:** 1 Department of Cardiac Thoracic Vascular Sciences and Public Health, University of Padova, 35128 Padova, Italy; andrea.fiorencis@gmail.com (A.F.); marikamrt91@gmail.com (M.M.); valeria.pergola@gmail.com (V.P.); marco.previtero@aopd.veneto.it (M.P.); sabino.iliceto@unipd.it (S.I.); 2 Department of Cardiology and Cardiac Surgery, S. Michele Hospital, 81024 Maddaloni, Caserta, Italy; marcpep@hotmail.it (M.P.); dr.sseverino@gmail.com (S.S.); doc.evangelista@gmail.com (M.E.); pierluigiincarnato@hotmail.it (P.I.); 3 Cardiology Unit, Umberto I Hospital, 48022 Lugo, Ravenna, Italy; vittoriosmarrazzo@hotmail.it; 4 GLM Esaote Spa, 16152 Genova, Italy; marco.maglione@esaote.com; 5 Department of Engineering and Architecture, University of Trieste, 34127 Trieste, Italy; giannip@diap.units.it

**Keywords:** intracardiac flow dynamics, vortex flow, kinetic energy dissipation, HyperDoppler, athletes

## Abstract

Background: HyperDoppler is a new echocardiographic color Doppler-based technique that can assess intracardiac flow dynamics. The aim of this study was to verify the feasibility and reproducibility of this technique in unselected patients and its capability to differentiate measures of vortex flow within the left ventricle (LV) in normal sedentary subjects, athletes, and patients with heart failure. Methods: Two hundred unselected, consecutive patients presenting at the echocardiographic laboratory, 50 normal subjects, 30 athletes, and 50 patients with chronic heart failure and LV ejection fraction <50% were enrolled. Images were acquired using a MyLab X8 echo-scanner. Area, intensity, depth, length, and kinetic energy dissipation (KED) of vortex flow were measured. Results: The HyperDoppler technique feasibility was 94.5%. According to the intraclass correlation coefficient evaluations, repeatability and reproducibility of vortex flow measures were good for vortex area (0.82, 0.85), length (0.83, 0.82), and depth (0.87, 0.84) and excellent for intensity (0.92, 0.90) and KED (0.98, 0.98). Combining different vortex flow measures, the LV flow profile of healthy sedentary individuals, athletes, and heart failure patients could be differentiated. Conclusions: HyperDoppler is a feasible, reliable, and practical technique for the assessment of LV flow dynamics and may distinguish normal subjects and patients with heart failure.

## 1. Introduction

Assessment of cardiac function using echocardiography is generally based on the evaluation of cardiac mechanics and transvalvular flow velocities. However, cardiac function is also an expression of intracardiac flow dynamics, that is, the peculiar organization of flow in vortical structures which occurs within the cardiac cavities, especially the left ventricle (LV). Previous echocardiographic studies showed that two vortices can be recognized within the LV during diastole, one located anteriorly (with clockwise rotation) across the LV inflow–outflow region and the other one (with counterclockwise rotation) located posteriorly [1]. Vortex flow generally dissolves during the LV ejection. Vortical flow organization plays a physiological role [1,2], and disturbances of intracardiac flow dynamics have been reported to have a negative impact on cardiac function [3].

Evaluation of intracardiac flow dynamics has been limited by the need to use phase-contrast cardiac magnetic resonance (PC-CMR) or contrast echocardiography with particle imaging velocimetry (echo-PIV), which are impractical approaches for an extensive application in patients [4,5,6]. In the last years, color Doppler-based ultrasound techniques have been developed to analyze organized vortical structures in the heart in a more practical way. HyperDoppler and Vector Flow Mapping (VFM) [7] are two of these techniques.

HyperDoppler relies on a different conceptual and technical approach compared with VFM, provides several geometrical and energy measures of vortical flow in addition to visual evaluation, and has the potential for extensive clinical utilization. There is a number of questions, however, to be answered to ascertain the validity and reliability of the HyperDoppler technique before proposing it for clinical applications: (1) Is the normal behavior of flow dynamics within the LV accurately recognized by this technique, according to physiology?; (2) What is the feasibility, repeatability, and reproducibility of quantitative measures?; (3) Are quantitative HyperDoppler flow measures different among individuals with expected differences in intracardiac flow dynamics, such as normal sedentary subjects, athletes, and patients with cardiac disease? In this study, we sought to answer these questions.

## 2. Material and Methods

### 2.1. Study Subjects and Patients

This is an observational multicenter study that involved two Italian cardiology Centers: the Cardiology Clinic of the Padova University Hospital in Padova (Center 1) and the Cardiology Clinic of the S. Michele Hospital in Maddaloni (Center 2). Each center was asked to enroll 100 unselected, consecutive patients presenting at the echocardiography laboratory for a clinical examination, regardless of the indication to echocardiography. At each center, the feasibility, repeatability (re-evaluation of the same individuals by the same observer), and reproducibility (evaluation of the same individuals by a different observer) of the HyperDoppler technique were tested. The repeatability and reproducibility of the two centers were compared to assess the intercenter differences. In addition, each center recruited 25 normal subjects, and Center 2 also recruited 30 athletes (enrolled consecutively among those presenting at the sports medicine clinic for a routine echocardiographic examination) and 50 patients with stabilized chronic heart failure and LV ejection fraction (EF) <50% (enrolled consecutively among those presenting at the heart failure clinic for programmed echocardiography). These latter 3 groups of individuals were compared to test the capability of the HyperDoppler technique to recognize significant differences in LV flow dynamics.

For all groups, the inclusion criterion was age >18 years. Exclusion criteria were decompensated acute heart failure and pregnancy. Patients with atrial fibrillation were not excluded from the study. Normalcy was defined by the absence of a history of any cardiac, renal, lung, metabolic, and blood disease and by normal echocardiography. Athletes were all professional football players. 

### 2.2. Standard Echocardiographic Examination

Transthoracic two-dimensional (2D) and Doppler echocardiographic examinations (including tissue Doppler) were carried out with a MyLab X8 echo scanner equipped with a 1–5 MHz electronic phased-array transducer (Esaote, Firenze, Italy). Images were acquired with the subjects and patients in the left lateral decubitus position at hold end-expiration. Trained physicians did all the echocardiographic measures according to the recommendations of the American Society of Echocardiography/European Association of Cardiovascular Imaging [8]. For each Doppler-based and M-mode measurement, estimates were obtained from 3 cardiac cycles in sinus rhythm or 5 in atrial fibrillation.

LV end-diastolic diameter, interventricular septum, and posterior wall thickness were measured on the parasternal long-axis view using the leading edge-to-leading edge approach. LV volumes and ejection fraction were calculated from the apical 4- and 2-chamber views using the biplane Simpson’s method. Assessment of LV diastolic function was made following the currently recommended algorithm, which includes mitral inflow pattern (peak E-wave/peak A-wave ratio, E/A), average peak E wave/peak e’ wave (E/e’) ratio, left atrial maximal volume index and peak tricuspid regurgitation velocity [9]. Cardiac valve regurgitations were graded to conform to current guidelines [10]. The tricuspid annular plane systolic excursion was measured using M-mode echocardiography in the apical 4-chamber view to determine the right ventricular longitudinal function.

The Mosteller formula for body surface area was used for indexation. Blood pressure, heart rate, and rhythm were recorded at the time of the echocardiographic examination.

### 2.3. HyperDoppler Technique Description

The complete 2D velocity vector flow field is recovered by the HyperDoppler technique on the basis of fluid dynamics concepts. A full description of the technique is reported in Appendix A.

### 2.4. HyperDoppler Image Acquisition

The HyperDoppler image acquisition was performed using the same MyLab X8 echo scanner and cardiac probe used for the conventional examination, with subjects and patients in the left lateral decubitus position, as previously described. A standard apical color Doppler long-axis view was acquired at hold end-expiration in a cineloop format, including two consecutive cardiac cycles, and stored in the echo scanner for the analysis. Depth and sector width were set to achieve a color Doppler frame rate ≥21 fps. Particular care was taken to include as much as possible of the LV cavity and the LV outflow tract (LVOT) within the color Doppler sector angle. Color Doppler pulse repetition frequency was 4.4 MHz. In each center, the first operator repeated the acquisition of the apical color Doppler long-axis view at the end of the echocardiographic examination; then, a second operator performed the same acquisition. Both the echo scanner setting and the images acquired by the first operator were unknown to the second one, who performed the acquisition in a blinded way.

### 2.5. HyperDoppler Image Analysis

At the end of the echocardiographic examination, each observer performed the analysis of the color Doppler cineloops on board the echo scanner. First, stored image cineloops were retrieved. Then, a region of interest was traced on the endocardial borders to include the LV cavity at end-diastole. The mitral annular plane and the LVOT were identified using a straight line. For the LVOT, the line was traced approximately 5 to 10 mm from the aortic valve plane. Finally, the vector velocity recovering algorithm was launched. The automatic output of this algorithm was represented by: (1) the velocity vector map; (2) a steady streaming flow field map; and (3) a table with the values of quantitative LV flow measures (described below).

Visual analysis. On the velocity vector map, temporal variations can be followed frame by frame, allowing the visual analysis at specific points in time. In normal subjects, this analysis was directed at recognizing the presence of the anterior and posterior vortex in the LV during early diastole, diastasis, late diastole, and isovolumetric contraction.

**Figure 1:**
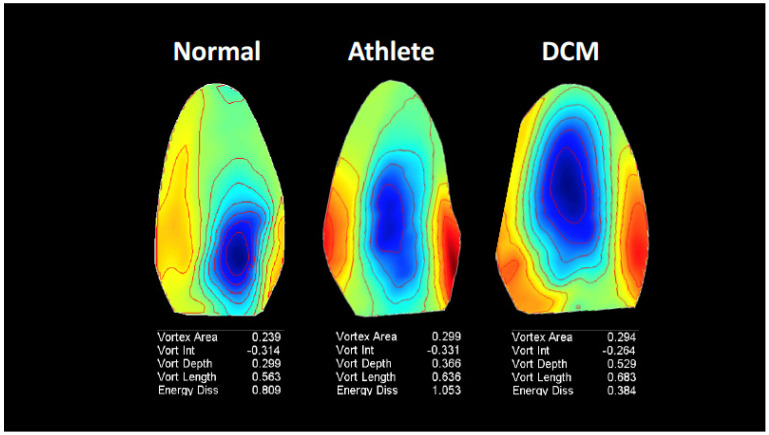
Examples of steady-streaming flow images of the left ventricle of different individuals. The steady-streaming flow field evaluates the overall circulatory pattern in the left ventricle during one heartbeat. DCM—dilated cardiomyopathy.

Quantitative flow parameters. Five scalar dimensionless measures related to the flow pattern were used, as previously described [11], to summarize vortex flow properties in the whole LV and over the whole heartbeat. To obtain measures characterizing the geometry and position of the vortex, the steady streaming (heartbeat average) flow field was computed to evaluate the overall circulatory pattern in the LV during one heartbeat. This picture can be considered as a sort of fingerprint of the LV flow (Figure 1) [1].

In this single image, the extension of the net circulatory region during one heartbeat is visualized. On the basis of this image, the fundamental intraventricular vortex is defined as the compact region about the steady streaming vortex center, where the stream function is larger than one-half of its peak value at the vortex center. The geometrical vortex properties of this net circulatory region are expressed by the following measures: the vortex area, normalized with the LV area; the vortex intensity (i.e., the integral of the vorticity inside the vortex), normalized with the total vorticity; the vortex depth (the distance of its center from the LV base) and the vortex length along the base-apex direction, both normalized with the LV length. The energetic properties of the vortex flow are evaluated by calculating the total kinetic energy dissipation (KED), that is, the amount of kinetic energy dissipated into the heart (by viscous friction) during the cardiac cycle [11,12]. The total KED is the value integrated over the entire LV; it is usually normalized with the average kinetic energy to avoid direct dependence on the LV size [11,12]. In the first 15 unselected patients at Center 1, the time needed for vortex analysis was measured as the time from starting to recall the stored color Doppler cineloop for analysis to the analysis output.

### 2.6. Effect of Measurement Variations

Two measurement variations were performed to assess the reliability of the approach used for vortex analysis: (1) To evaluate the variability of vortex flow measures related only to LV border tracing, at Center 1 the second observer reanalyzed the images acquired by the first observer in 10 patients randomly extracted from the unselected patient group. Results were compared with those obtained including both image acquisition and analysis in the same patients; (2) To test the effect of including the entire LVOT within the LV border, at Center 1 the first observer delineated the LVOT with a straight line at the level of the aortic valve plane in 10 patients randomly extracted from the unselected patient group (these patients are different from those of point 1). Results were compared with those obtained with the measurement of the LVOT as previously described, performed by the same observer in the same patients.

### 2.7. Statistical Analysis

Normal distribution was tested with the Kolmogorov–Smirnov test. Continuous variables were reported as mean ± standard deviation and categorical variables as counts and percentages. For continuous variables, the one-way ANOVA and Student’s *t*-test were used for global and pairwise comparisons, respectively. Categorical variables were compared by the chi-square test. The feasibility of the HyperDoppler technique was calculated as the percentage of patients with color Doppler image quality and frame rate suitable for vortex analysis with respect to the total of the patients. The repeatability and reproducibility of vortex measures at each Center were assessed using the intraclass correlation coefficient (ICC) and the limits of agreement (LOA) at the Bland–Altman analysis. An ICC <0.50 was considered poor, between 0.50 and 0.74 moderate, between 0.75 and 0.89 good, and ≥0.90 excellent. For each vortex flow measure, the intercenter difference was assessed by calculating the ΔICC, which was reported as absolute difference and percentage (obtained by indexing the Δ by the Center 1 value). A variation of <10% was considered indicative of low and <5% of very low intercenter variability. Data were analyzed using MedCalc (MedCalc Software Ltd., Ostend, Belgium), v. 15.8. A *p* value < 0.05 was considered statistically significant. The Bonferroni correction was applied in case of multiple comparisons.

## 3. Results

Anthropometric, clinical, and echocardiographic characteristics of unselected patients, normal subjects, athletes, and patients with heart failure and LV-EF < 50% are reported in Table 1. Among patients with heart failure, 18 had an LV EF between 40 and 49% (mildly reduced), 24 had an LV EF between 30% and 39%, and 8 had an LV EF <30% (severely reduced). Characteristics of the unselected, consecutive patients examined at Centers 1 and 2 were similar.

### 3.1. Feasibility

Among unselected, consecutive patients, vortex flow analysis could not be performed in 5 (5%) patients at Center 1 and in 6 (6%) patients at Center 2 because of inadequate color Doppler image quality or insufficient frame rate. Thus, overall feasibility was 94.5%. The time needed for quantitative analysis of acquired images was 49 ± 9 s.

### 3.2. Descriptive Analysis

**Figure 2:**
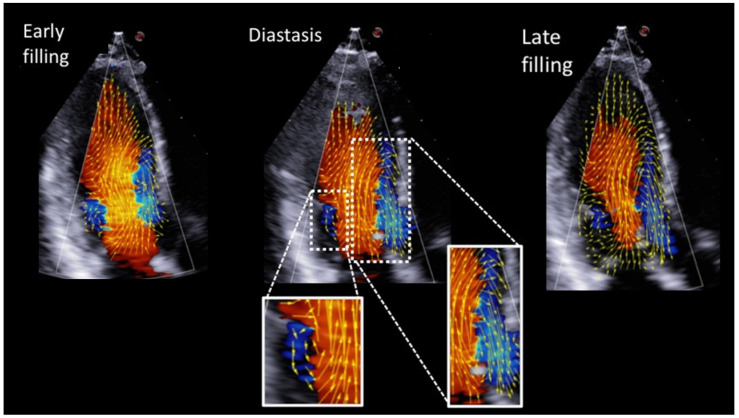
Apical long-axis views with flow velocity vector maps showing mitral inflow during early filling, diastasis and late filling with a pair of counterrotating vortices, one distal to the anterior mitral valve leaflet (larger vortex) and one distal to the posterior leaflet (smaller vortex). Velocity vectors are overimposed on the standard color Doppler flow representation. The anterior and posterior vortices are magnified for better appreciation.

Intraventricular flow dynamics could be analyzed visually in all the 50 healthy subjects. An example of a normal subject is shown in Figure 2. The results of the descriptive analysis are summarized in Table 2. In the vast majority of normal subjects, two vortices were recognized at early diastole; the anterior one was generally larger than the posterior one. During diastasis, the anterior vortex was still seen in the majority of patients, whereas the posterior vortex was observed less frequently. At the moment of atrial systole, the anterior and posterior vortex were again both visible in most subjects, whereas during the isometric contraction time, only the anterior vortex was evident. Vortical flow disappeared during the ejection phase.

### 3.3. Repeatability and Reproducibility

According to the ICC values, at Center 1 repeatability and reproducibility of vortex flow measures in unselected patients were good for vortex area (0.82, 0.85), length (0.83, 0.82), and depth (0.87, 0.84) and excellent for vortex intensity (0.92, 0.90) and KED (0.98, 0.98) (Table 3 and Table 4). Similar results were observed in the subgroup of patients with atrial fibrillation (Supplementary Materials Tables S1 and S2).

Results of the Bland–Altman analysis are graphically displayed in Supplementary Materials Figures S1 and S2. No bias (consistent under or overestimation) was observed for each vortex flow measure.

### 3.4. InterCenter Variability

Center 2 provided comparable repeatability and reproducibility evaluations for all the vortex flow measures (Table 3 and Table 4, Supplementary Materials Figures S3 and S4). Intercenter differences in vortex flow measures were <5% of the ICC for repeatability and <8% of the ICC for reproducibility. Similar results were observed in the subgroup of patients with atrial fibrillation (Supplementary Materials Tables S1 and S2).

### 3.5. Effect of Measurement Variations

Including the entire LVOT within the LV produced a slightly significant increase in KED, whereas geometrical and intensity vortex flow measures did not vary (Table 5). Repeated LV border tracing did not significantly change vortex flow measures (Table 5).

### 3.6. Quantitative Analysis

Values of flow measures that quantitatively describe vortex characteristics in healthy sedentary subjects, athletes, and patients with heart failure are reported in Table 6. All healthy subjects and athletes could be analyzed, whereas vortex analysis was possible in 47 out of the 50 heart failure patients (94%). Overall, all the vortex flow measures were different across the three groups of individuals but with specific differences in the pairwise comparisons (Table 6). Athletes had greater vortex area, intensity, and KED compared with healthy sedentary subjects, while they had smaller vortex depth and length and greater KED compared with patients with heart failure and reduced LV-EF (Table 6). In comparison with healthy subjects, heart failure patients showed greater vortex area, length, depth, and intensity but smaller KED (Table 6).

## 4. Discussion

In this paper, we provide visual evaluations and quantitative estimates of flow measures relative to the application of the new HyperDoppler technique to the study of intracardiac flow dynamics in normal sedentary subjects, athletes, and patients with heart failure. We observed that this technique: (1) is highly feasible, repeatable, and reproducible; (2) allows the description of flow dynamics physiology within the LV of normal individuals; and (3) can differentiate normal sedentary subjects, athletes, and patients with heart failure and reduced LV-EF using quantitative measures of LV vortex flow. Thus, this technique is reliable for application in humans and suitable for testing pathophysiological and clinical hypotheses.

### 4.1. Study of Left Intraventricular Flow Dynamics

Evaluation of cardiac function is a key issue for all cardiac imaging techniques, in addition to morphological, structural, and hemodynamic evaluations. Generally, the heart function is studied by observing the motion and deformation of the myocardial walls or the velocities of transvalvular flows. A different way to approach the study of cardiac function is the analysis of intracardiac flow dynamics. For the past 20 years, PC-CMR and echo-PIV have been used for this purpose, providing a range of physiological and pathological observations [4,5,6]. However, these techniques have not entered clinical practice, mainly because they have limited availability, are impractical (complex and time-consuming), and require specific operator skills. In addition, the need for ultrasound contrast makes the application of echocardiography to the study of intracardiac flow dynamics unfeasible in most ambulatory settings and expensive. More recently, color Doppler-based ultrasound techniques have been introduced, which allow to reconstruct the velocity vector field in a practical, semiautomated, and contrast-free way [13,14,15]; hence, there are renewed expectations on the possibility of bringing intracardiac vortex assessment into routine clinical practice. An advantage of these color Doppler-based ultrasound techniques is that velocity accuracy is good, because it is that of Doppler. A general limitation is that the velocity vector is reconstructed, not measured; thus, anomalous phenomena may remain hidden.

### 4.2. HyperDoppler vs. Other Color-Doppler Based Techniques

VFM (Hitachi, Aloka Medical Ltd., Tokyo, Japan) is an ultrasound technique that, like HyperDoppler, evaluates intracardiac flows using a velocity vector reconstruction algorithm based on color Doppler [7]. Differences between these two techniques should be clearly understood since they are the only two-color Doppler-based ultrasound techniques commercially available for current clinical application. These differences essentially rely on the approach to the reconstruction of the velocity vector field and the measures used for flow quantitation. As far as the first point is concerned, VFM is based on a speckle tracking approach to obtain the values of transversal flow velocities [7]. This is explained in more detail in the Appendix A. Regarding flow measures, they are quantitated as absolute values by VFM, whereas they are normalized by the whole LV and over the whole heartbeat by the HyperDoppler technique, thus generating dimensionless indexes. Whether measures obtained by the two techniques are comparable remains to be established by a side-by-side comparison in the same normal subjects and patients.

### 4.3. Reliability of the HyperDoppler Technique

In this study, the feasibility of the HyperDoppler technique was excellent, and the time for analysis was relatively short. Overall, repeatability and reproducibility were good-to-excellent, although there were some differences in ICC among vortex flow measures. For example, repeatability and reproducibility of geometrical vortex measures were good, whereas those of KED were excellent (Table 3 and Table 4). This, in our opinion, shows that KED is probably less sensitive to variability in image acquisition compared with geometrical flow measures. Intercenter variability (same type of echo-scanner, different unselected patients, different observers) was low to very low since Center 2 generally provided comparable repeatability and reproducibility with respect to Center 1 for all vortex flow measures, especially for KED (Table 3 and Table 4). These results highlight the robustness of the HyperDoppler technique.

In this study, observer variability included both image acquisition and analysis. Thus, in a subgroup of 10 patients we separately assessed interobserver variability due to image analysis only. Since this latter variability was negligible (Table 5), we can deduce that overall observer variability is essentially related to image acquisition, which, in turn, is probably more dependent on differences in image plane orientation than on variations in machine settings. Indeed, standard criteria for setting the echo scanner were used by all the operators; thus, it is unlikely that machine setting could have had any substantial impact on the variability of acquired images.

In this investigation, we also tested the effect of tracing the LVOT at the level of the aortic valve plane. This approach did not change significantly geometrical vortex measures and vortex intensity compared to the LVOT delineation approximately 5 to 10 mm from the aortic valve plane. However, it slightly but significantly increased KED (Table 5). This is reasonable since the LVOT generally includes the highest LV systolic flow velocities and thus the highest KED. To avoid the effect of these outflow velocities when the KED of the main LV cavity is evaluated, we suggest performing the LVOT tracing below the aortic valve plane.

### 4.4. Visual Evaluation of Vortex Flow in Normal Subjects

In normal subjects, the HyperDoppler technique was able to recognize visually vortex physiology (Figure 2). Overall, the anterior vortex was observed during diastole in a very high percentage of cases (Table 2); the posterior vortex was also very frequent during early and late diastole, while it was much less evident during diastasis (Table 2). Our findings are in agreement with previous observations reported using different techniques [11,16]. Interestingly, Elbaz et al. [16] attributed the lack of the LV vortex during atrial systole in some patients to a higher heart rate and limited diastasis duration, which might not allow the development of the LV pressure gradient required for vortex formation. In our study, however, we could not confirm this hypothesis.

### 4.5. Quantitative Evaluation of Vortex Flow

Flow measures obtained using the HyperDoppler technique allowed a quantitative description of the size, position, and KED of the vortex structure within the LV (Table 6). 

In normal sedentary subjects, vortex length values (0.62 ± 0.11) indicate that the vortex structure normally extends from base to apex for most of the LV. The vortex area was, on average, 0.26, with a relatively small standard deviation (0.05), suggesting a quite consistent area size. Vortex position, as indicated by vortex depth (0.33 ± 0.08), was also consistent. These results are similar to those reported by Cimino et al. [11] using the echo-PIV technique with contrast administration. Conversely, KED had a high standard deviation (0.35), which indicates a higher variability in comparison with geometrical vortex features.

Athletes showed higher values of vortex area compared with normal sedentary subjects, although with similar vortex length. Vortex depth did not show any difference compared to normal subjects, indicating that vortex area, although increased, respects physiological vortex position within the LV. Vortex intensity was increased, most likely because of increased vortex area (vortex intensity depends directly on area). KED was markedly increased. This probably relates to the higher flow velocities of early LV filling and is in line with the observations of Steding-Ehrenborg et al. [17], who reported that kinetic energy at early diastole is higher in athletes compared with control subjects, indicating enhanced diastolic function.

In patients with heart failure and dilated and dysfunctioning LV, the vortex flow profile was characterized by greater vortex area and length compared with normal sedentary subjects. Depth was also greater, indicating an anomalous location of the bigger vortex, which is displaced towards the apex. This geometrical vortex profile differentiates from that of athletes, who showed a similarly increased vortex area but with a physiological location. Vortex intensity was also increased in heart failure patients with reduced LV-EF, most probably as a consequence of the greater vortex area (as previously pointed out for athletes). KED behavior was the most striking one since it decreased markedly with respect to both normal sedentary subjects and athletes. We speculate that this finding relates to reduced kinetic energy, which in these patients is very likely affected by the reduction of both the LV early peak filling velocity and force of contraction. These findings agree with the observations of Mangual et al. [18], who reported small values of KED in patients with dilated cardiomyopathy (throughout the cardiac cycle and separately during systole and diastole) in comparison with normal subjects. They also agree with the results of Elbaz et al. [19], who described a direct relationship between kinetic energy and KED.

### 4.6. How to Approach Vortex Analysis in Practice

The aforementioned data suggest that HyperDoppler vortex analysis can be used to describe cardiac physiology and pathology through a combination of geometrical and energetic flow measures. The location of the main vortex, indicated by vortex depth, differentiates normal physiology from a pathological condition, regardless of vortex size. Vortex area can be increased in athletes; thus, it does not necessarily mean a pathological status. The same applies to vortex intensity since it depends directly on the vortex area. KED represents a very interesting measure because it may vary regardless of geometrical vortex measures. In summary, it is evident that the information coming from vortex size, location (depth), and KED should be integrated for a correct understanding of intracardiac flow dynamics. Looking at only one of these markers can be misleading or at least provide only a partial view of the whole vortex flow picture.

### 4.7. Study Advantages

This study has several advantages. Because of the number of patients used for repeatability and reproducibility analysis, results may be considered stabilized. The wide range of values obtained in unselected patients for all the LV flow measures allowed a reliable Bland–Altman analysis. All the main vortex flow measures were considered and tested in terms of both repeatability/reproducibility and comparison among normal sedentary subjects, athletes, and patients with heart failure. We examined true observer variability, which included variability for both image acquisition and analysis. Analysis of LV flow dynamics was conducted on-board of the echo scanner, according to what generally occurs in clinical echocardiographic practice.

### 4.8. Study Limitations

In this study, intracardiac flow dynamics assessed using the HyperDoppler technique was not compared with evaluations obtained with other techniques (i.e., PC-CMR or echo-PIV). This could be the goal of a dedicated investigation. However, because both PC-CMR [20,21] and echo-PIV [22] have their own well-characterized limitations in the measurement of intracardiac flow velocity, a head-to-head analysis should not be interpreted as a formal validation study.

This study was not directed at identifying patterns of flow or vortex quantitative measures in specific cardiac diseases but at evaluating the capability of the HyperDoppler technique to differentiate individuals with expected differences in intracardiac flow dynamics. Further studies are needed to address the diagnostic, prognostic, and treatment-guiding value of this technique in single cardiac diseases.

The athlete group included only males because an entire male football team was evaluated. This makes this group of subjects very homogeneous because the intensity and type of training were the same. Further studies are needed to investigate the effect of different types of sports on intracardiac flow dynamics.

The vortex analysis was performed only using one LV apical view, namely the long-axis view, because it contains both the mitral inflow and the aortic outflow tract. It is therefore limited to a 2D assessment of the actual 3D flow field. Additional studies are needed to explore the analysis of LV flow dynamics in other LV apical views or by 3D Doppler echocardiography [23]. For the VFM technique, some authors reported that this method is most accurate for recordings in transthoracic echocardiography when the scan plane contains both mitral and aortic valves and the apex (apical 3- and 5-chamber views) [14,15].

Finally, this study did not intend to evaluate the physiological determinants of intracardiac flow dynamics as assessed by the HyperDoppler technique, including age and gender. Other investigations using the VFM technique for vortex analysis reported an effect of age on diastolic intracardiac flow patterns [24,25]. In our study, the differences in vortex characteristics between normal sedentary subjects, athletes, and patients with heart failure could have been affected by differences in age and gender.

## 5. Conclusions

HyperDoppler is a new ultrasound technique that is reliable and practical for the assessment of LV flow dynamics. It can quantitate several measures of the LV vortex and may distinguish normal sedentary subjects, athletes and patients with heart failure. Future studies are needed to clarify how to implement this technique in cardiology clinical practice.

## Figures and Tables

**Table 1:** Characteristics of normal subjects, athletes, and patients. A—absent or trivial; AR—aortic regurgitation BSA—body surface area; DBP—diastolic blood pressure; DCM—dilated cardiomyopathy; EDD—end-diastolic diameter; EDT—end-diastolic thickness; EF—ejection fraction.; F—female; HHD—hypertensive heart disease; IHD—ischemic heart disease; IVS—interventricular septum; LAVi—left atrial volume index; LV—left ventricle; M—male; Mi—mild; Mo—moderate; MR—mitral regurgitation; PW—posterior wall; S—severe; SBP—systolic blood pressure; TAPSE—tricuspid annulus plane systolic excursion; VHD—valve heart disease.

	Unselected Patients			Normal Subjects	Athletes	Heart Failure Patients	
	Center 1	Center 2	*p* Value	Centers 1 and 2	Center 2	Center 2	*p* Value
Individuals (n)	100	100	-	50	30	50	-
Age (years)	65 ± 16	62 ± 16	0.24	37 ± 13	24 ± 5	69 ± 11	<0.001
Sex (M/F)	67/33	56/44	0.11	23/27	30/0	29/21	<0.001
BSA (m^2^)	1.83 ± 0.21	1.84 ± 0.20	0.72	1.7 ± 0.2	1.96 ± 0.12	1.83 ± 0.23	<0.001
Heart rate (bpm)	74 ± 14	73 ± 9	0.40	62 ± 6	56 ± 7	76 ± 8	<0.001
SBP (mmHg)	128 ± 13	131 ± 12	0.82	126 ± 11	114 ± 5	116 ± 15	<0.001
DBP (mmHg)	77 ± 8	80 ± 9	0.43	78 ± 5	73 ± 5	70 ± 10	<0.001
IHD (n)	21	17	0.108	0	0	10	-
HHD (n)	31	26		0	0	5	-
VHD (n)	12	14		0	0	3	-
DCM (n)	11	9		0	0	24	-
Other etiologies (n)	25	34		0	0	8	-
Atrial fibrillation (n)	13	11	0.663	0	0	3	-
LV-EDD (cm)	4.8 ± 0.8	4.7 ± 0.7	0.81	4.5 ± 0.4	5.2 ± 0.3	6.7 ± 0.7	<0.001
IVS-EDT (cm)	1 ± 0.2	1 ± 0.2	0.12	0.8 ± 0.1	0.9 ± 0.2	1 ± 0.2	<0.001
PW-EDT (cm)	1 ± 0.2	1 ± 0.1	0.20	0.7 ± 0.1	0.9 ± 0.1	1 ± 0.2	<0.001
LV-EDVi (mL/m^2^)	57 ± 26	52 ± 20	0.12	51 ± 8	63 ± 9	84 ± 25	<0.001
LV-ESVi (mL/m^2^)	27 ± 22	25 ± 15	0.42	21 ± 4	27 ± 5	60 ± 23	<0.001
LV-EF (%)	57 ± 13	56 ± 10	0.66	60 ± 4	61 ± 3	37 ± 8	<0.001
Peak E-wave (cm/s)	76 ± 25	72 ± 19	0.22	75 ± 15	87 ± 12	70 ± 19	<0.001
Peak A-wave (cm/s)	70 ± 28	73 ± 23	0.51	57 ± 17	54 ± 14	74 ± 32	<0.001
E/A ratio	1.4 ± 1.2	1.6 ± 0.5	0.63	1.4 ± 0.5	1.6 ± 0.5	1.1 ± 0.7	<0.001
Peak e’ (cm/s)	9 ± 3	10 ± 7	0.85	13 ± 3	16 ± 3	5 ± 2	<0.001
E/e’ ratio	10 ± 7	8 ± 3	0.13	6 ± 1.7	5 ± 1.2	15 ± 7	<0.001
LAVi (mL/m^2^)	33 ± 18	32 ± 16	0.76	19 ± 4	32 ± 6	31 ± 13	<0.001
TAPSE (cm)	2.4 ± 0.5	2.5 ± 0.4	0.15	2.7 ± 2.8	2.6 ± 0.4	2.0 ± 0.4	<0.001
MR (A/Mi/Mo/S)	26/58/15/1	27/52/19/2	0.284	50/0/0/0	30/0/0/0	3/29/12/6	<0.001
AR (A/Mi/Mo/S)	55/31/13/1	60/27/10/3	0.17	50/0/0/0	30/0/0/0	42/5/2/1	0.03
Pericardial effusion	1	0	0.316	0	0	1	0.204

**Table 2:** Visual recognition of the anterior (A) and posterior (P) vortex within the left ventricle during the cardiac cycle in 50 normal subjects. N % = number and percentage of patients in whom the vortex is recognized.

	Early Filling		Diastasis		Late Filling		Isometric Contraction
	A-Vortex	P-Vortex	A-Vortex	P-Vortex	A-Vortex	P-Vortex	A-Vortex
N	48	44	39	15	46	37	45
%	96	88	78	30	92	74	90

**Table 3:** Repeatability of vortex flow measures in unselected consecutive patients. ICC—intraclass correlation coefficient; LOA—limits of agreement. ICC is reported with a 95% confidence interval (in parentheses). ΔICC is reported as absolute difference and percentage (obtained by indexing Δ by the Center 1 value). KED—kinetic energy dissipation.

	Repeatability		
	Center 1 (N = 95)	Center 2 (N = 94)	Center 2 vs. Center 1
Vortex area	ICC = 0.86 (0.79–0.90)	ICC = 0.88 (0.83–0.92)	ΔICC = 0.02 (2.2%)
	LOA = 0.07, −0.06	LOA = 0.06, −0.05	
Vortex length	ICC = 0.85 (0.78–0.90)	ICC = 0.82 (0.74–0.88)	ΔICC = 0.03 (3.5%)
	LOA = 0.12, −0.11	LOA = 0.11, −0.0	
Vortex depth	ICC = 0.88 (0.83–0.92)	ICC = 0.91 (0.87–0.94)	ΔICC = 0.03 (3.4%)
	LOA = 0.08, −0.09	LOA = 0.07, −0.08	
Vortex intensity	ICC = 0.92 (0.89–0.95)	ICC = 0.90 (0.85–0.93)	ΔICC = 0.02 (2.2%)
	LOA = 0.06, −0.07	LOA = 0.06, −0.07	
KED	ICC = 0.98 (0.96–0.98)	ICC = 0.98 (0.97–0.99)	ΔICC = 0
	LOA = 0.14, −0.11	LOA = 0.14, −0.12	

**Table 4:** Reproducibility of vortex flow measures in unselected consecutive patients. ICC—intraclass correlation coefficient. LOA—limits of agreement. ICC is reported with a 95% confidence interval (in parentheses). ΔICC is reported as absolute difference and percentage (obtained by indexing Δ by the Center 1 value). KED—kinetic energy dissipation.

	Reproducibility		
	Center 1 (N = 95)	Center 2 (N = 94)	Center 2 vs. Center 1
Vortex area	ICC = 0.86 (0.80–0.91)	ICC = 0.86 (0.80–0.91)	ΔICC = 0.01 (1.2%)
	LOA = 0.08, −0.05	LOA = 0.07, −0.06	
Vortex length	ICC = 0.78 (0.70–0.85)	ICC = 0.78 (0.69–0.85)	ΔICC = 0.01 (1.2%)
	LOA = 0.12, −0.11	LOA = 0.12, −0.12	
Vortex depth	ICC = 0.91 (0.86–0.94)	ICC = 0.84 (0.78–0.89)	ΔICC = 0.05 (5.6%)
	LOA = 0.9, −0.06	LOA = 0.10, −0.09	
Vortex intensity	ICC = 0.94 (0.91–0.96)	ICC = 0.87 (0.81–0.91)	ΔICC = 0.07 (7.6%)
	LOA = 0.06, −0.07	LOA = 0.08, −0.07	
KED	ICC = 0.97 (0.96–0.98)	ICC = 0.97 (0.96–0.98)	ΔICC = 0
	LOA = 0.15, −0.14	LOA = 0.17, −0.16	

**Table 5:** Variability in vortex flow measures according to different LVOT measurements and repeated LV border tracing in 10 normal subjects. KED—kinetic energy dissipation.

	LVOT Measurement		*p* Value	Repeated LV Border Tracing		*p* Value
	Below the Aortic Valve Plane	At the Aortic Valve Plane		Observer 1	Observer 2	
Area	0.22 ± 0.07	0.22 ± 0.07	0.92	0.22 ± 0.07	0.22 ± 0.07	0.98
Depth	0.32 ± 0.08	0.31 ± 0.08	0.61	0.32 ± 0.08	0.32 ± 0.08	0.74
Length	0.53 ± 0.11	0.52 ± 0.11	0.91	0.53 ± 0.11	0.53 ± 0.11	0.98
Intensity	−0.27 ± 0.1	−0.27 ± 0.1	0.59	−0.27 ± 0.1	−0.27 ± 0.1	0.97
KED	0.90 ± 0.43	0.94 ± 0.43	0.04	0.82 ± 0.33	0.85 ± 0.45	0.99

**Table 6:** Vortex flow parameters values in different groups of subjects and patients. All values are reported as mean ± standard deviation. ATH—athletes; HF—heart failure; NS—normal subjects. KED—kinetic energy dissipation. For pairwise comparisons, a *p* < 0.017 was used for statistical significance (Bonferroni correction).

	Normal Subjects (N = 50)	Athletes (N = 30)	Heart Failure Patients (N = 47)	*p* Value
Vortex area	0.26 ± 0.05	0.31 ± 0.04	0.31 ± 0.04	*p* < 0.001 overall, NS vs. ATH and NS vs. HF
Vortex length	0.62 ± 0.11	0.67 ± 0.09	0.73 ± 0.08	*p* < 0.001 overall and NS vs. HF, *p* = 0.003 ATH vs. HF
Vortex depth	0.33 ± 0.08	0.32 ± 0.05	0.39 ± 0.06	*p* < 0.001 overall, NS vs. HF and ATH vs. HF
Vortex intensity	−0.35 ± 0.09	−0.41 ± 0.04	−0.42 ± 0.04	*p* < 0.001 overall, NS vs. ATH and NS vs. HF
KED	0.67 ± 0.35	1.23 ± 0.24	0.19 ± 0.09	*p* < 0.001 overall, NS vs. ATH, NS vs. HF and ATH vs. HF

## Data Availability

The data of the study are available upon justified request.

## Supplementary Materials

The following are available online at https://www.mdpi.com/article/10.3390/jcm11082216/s1, Figure S1: Bland-Altman analysis for vortex area, intensity and length on 95 unselected, consecutive patients examined at Center 1, Figure S2: Bland-Altman analysis for vortex depth and kinetic energy dissipation (KED) on 95 unselected, consecutive patients examined at Center 1, Figure S3: Bland-Altman analysis for vortex area, intensity and length on 94 unselected, consecutive patients examined at Center 2, Figure S4: Bland-Altman analysis for vortex depth and kinetic energy dissipation (KED) on 94 unselected, consecutive patients examined at Center 2, Table S1: Repeatability of vortex flow measures in patients with atrial fibrillation, Table S2: Reproducibility of vortex flow measures in patients with atrial fibrillation.

## Appendix A. Description of the HyperDoppler Technique

Consider a 2D image scan plane where the echographic apparatus reports the Doppler velocity at each point *X* of the plane *V_D_(X)*. The velocity is given at each frame time of the acquisition, and at least one image is necessary. The point coordinates *X* can be expressed in general as the two Cartesian coordinates *X = (x,y)* or the polar coordinates *X = (r,ϑ)*, where the radius *r* is the distance from the focus and *ϑ* is the sector angle, transversal to the radial direction.

The initial objective is the evaluation of the 2D velocity vector field *V(X)* in terms of Cartesian components *V_x_(X)*, *V_y_(X)*, or polar components *V_r_(X)*, *V_ϑ_(X)*. The polar coordinates are known by Doppler such that the radial velocity is simply *V_r_ = −V_D_*, and the transversal velocity *V_ϑ_* has to be computed. To this aim, the assumption of flow incompressibility on the plane is made, and the transversal coordinate can be computed by using the planar continuity equation, that in polar coordinates reads:

(A1) $$\frac{\partial rV_{r}}{\partial r}+\frac{\partial V_{\vartheta }}{\partial \vartheta }=0$$

Given that the radial velocity is known by the Doppler measurement in the echographic sector ranging from *ϑ*_1_ to *ϑ*_2_, the first term in the continuity Equation (1) can be computed, and the radial velocity is then evaluated using Equation (A1) by integration:

(A2) $$V_{\vartheta }\left(r,\vartheta \right)=-\int_{\vartheta_{0}}^{\vartheta }\frac{\partial rV_{r}}{\partial r}d\vartheta$$

where the integration can start from any position, here indicated by *ϑ*_0_, where the value of the transversal velocity is known. The integration Equation (A2) gives the solution apart from an undetermined function of the radial coordinate $V_{\vartheta }\left(r,\vartheta_{0}\right)$ and of time that must be specified with some assumption. Several methods propose different solutions to this problem (11); more recent proposals (13,14) suggest the integration of Equation (A2) along the two directions, and minimization of the errors in transversal velocity at the two ends, $V_{\vartheta }\left(r,\vartheta_{1}\right)$ and $V_{\vartheta }\left(r,\vartheta_{2}\right)$, by comparison to the values measured by speckle tracking. This approach considers the ground truth given by transversal speckle tracking values that are known to be subjected to large errors for local pointwise values. Moreover, the values at different radial positions are conceptually uncorrelated (apart from artificial smoothing), an assumption that is not realistic in physiological flows.

The present approach assumes that the velocity field is given by the Doppler component *V_D_(X)* plus an irrotational flow: *V(X) = V_D_(X)* + *U(X)*. Where the Doppler part is formally expressed as a vector built by the Doppler component only. The additional irrotational flow, *U(X)*, can be expressed as the gradient of a scalar potential *φ* as:

(A3) $$U\left(X\right)=\nabla \phi$$

The choice of an irrotational flow sometimes called potential flow, is due to the following reasons. A potential flow is the least disturbing flow in the sense that it does not modify the distribution of vorticity, which is the key quantity in any fluid flow; therefore, it does not significantly alter energy dissipation and shear stress distribution in the flow field. A potential flow does not enter, besides rigid transport terms, into the fluid dynamics energy balance. Nevertheless, the irrotational flow permits adjusting mass conservation; it can rather act on mass conservation only. Therefore, the choice of using an irrotational flow as a correction for continuity is the most natural choice from the fluid dynamics principle.

The potential flow permits to satisfy the continuity simply by applying the continuity equation that, in general, is expressed as the constraint of velocity having zero divergence:

(A4) ∇⋅V=0

that is equivalent to Equation (A1). The Doppler flow alone does not satisfy the continuity Equation (A4) and produces a spatial distribution of divergence. Application of the continuity Equation (A4) to the complete, Doppler plus potential flow gives the elliptic equation of Poisson type:

(A5) $$\nabla^{2}\phi =-\nabla \cdot V_{D}$$

where the right-hand side is a known term, computed from the Doppler velocity, and the potential *φ* is the unknown. Once the potential is obtained from the solution of Equation (A5), the total velocity can be computed by Equation (A3).

The elliptic Equation (A5) permits inserting any boundary condition that is physically consistent. It gives exactly the irrotational flow that is physically required to fulfill the continuity constraint and the desired boundary conditions.

The solution is here obtained in the Cartesian image coordinates. These allow the development calculation techniques that are particularly efficient and also ensure a more uniform distribution of errors or inaccuracies. The solution here is based on a spatial Fourier decomposition that allows fast Poisson solvers.

Moreover, in 2D imaging, the continuity equation is not necessarily exactly satisfied because of the cross-plane motion. In this study, we use the approach of solving Equation (A5), and apply the correction Equation (A3) to the transversal component only without correcting the radial velocity from the Doppler measurement. This solution is equivalent to assuming the presence of a cross-plane inflow/outflow that exactly replaces the neglected radial contribution.

The solution using this approach is the result of an elliptic equation; this means that all points of space are connected and that the solution presents a mathematical regularity. The solution is continuously differentiable in all directions at all points in space.
