# Supplementary material for: Noninvasive Evaluation of Intraventricular Flow Dynamics by the HyperDoppler Technique: First Application to Normal Subjects, Athletes, and Patients with Heart Failure
Source: J Clin Med. 2022 Apr 15;11(8):2216. doi: 10.3390/jcm11082216 (PMC9026209; doi:10.3390/jcm11082216)
Supplement: Supplementary file 1 [file jcm-11-02216-s001.zip › jcm-1653338-supplementary.pdf]

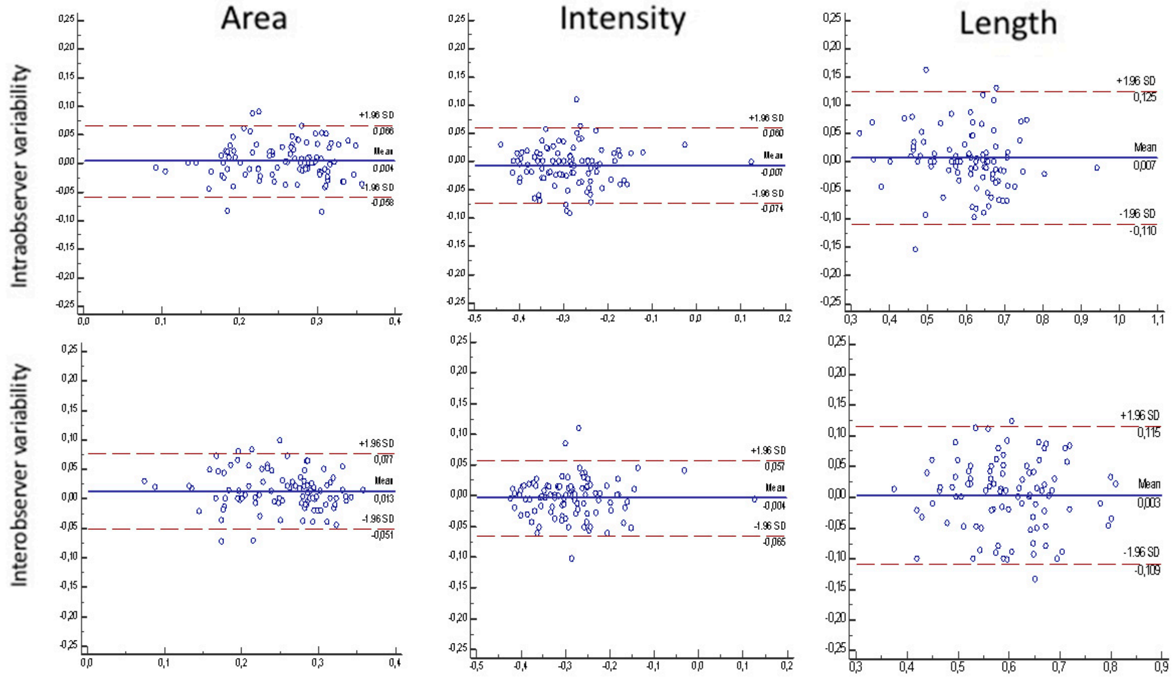

Figure S1. Bland-Altman analysis for vortex area, intensity and length on 95 unselected, consecutive patients examined at Center 1. In each graph, the abscissa shows the average value of the two measures whereas the ordinate shows the error (difference between the two measures). SD: standard deviation.

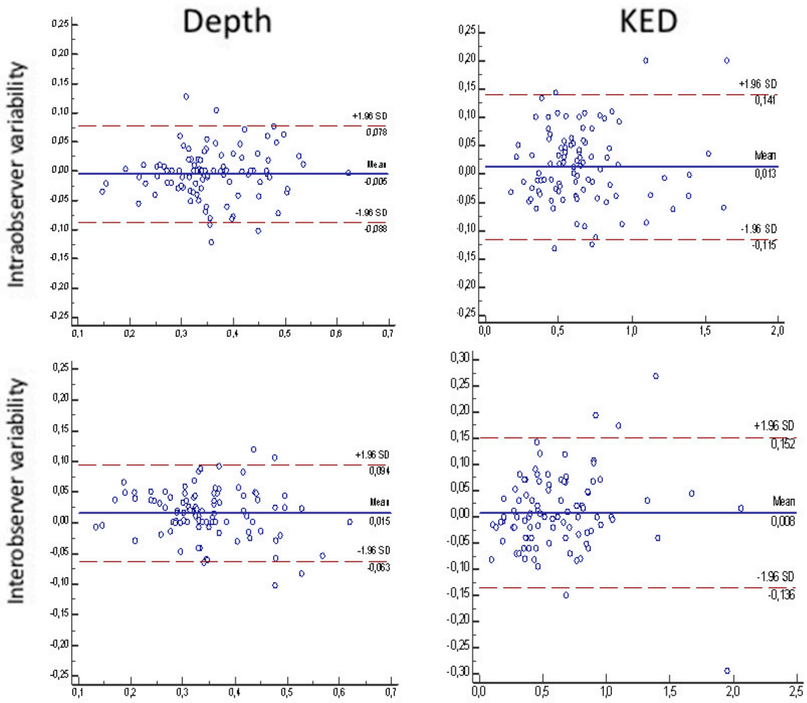

Figure S2. Bland-Altman analysis for vortex depth and kinetic energy dissipation (KED) on 95 unselected, consecutive patients examined at Center 1. Abscissa and ordinate measures as described in Supplemental Figure S1. SD: standard deviation.

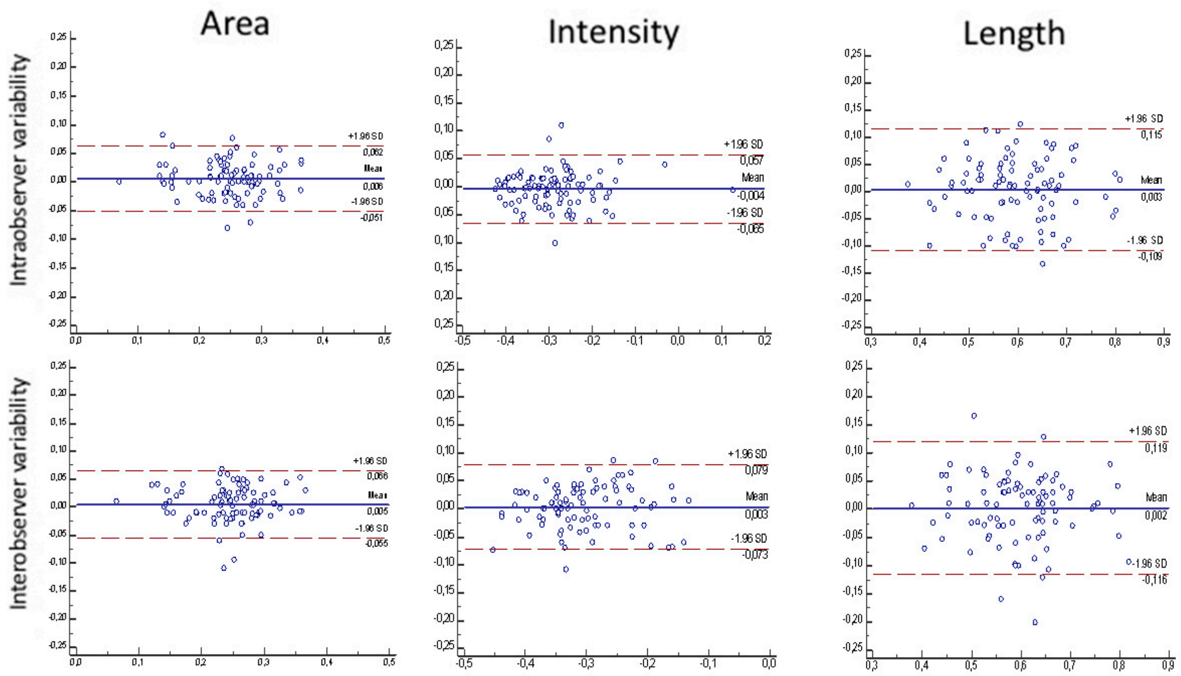

Figure S3. Bland-Altman analysis for vortex area, intensity and length on 94 unselected, consecutive patients examined at Center 2. Abscissa and ordinate measures as described in Supplemental Figure S1. SD: standard deviation.

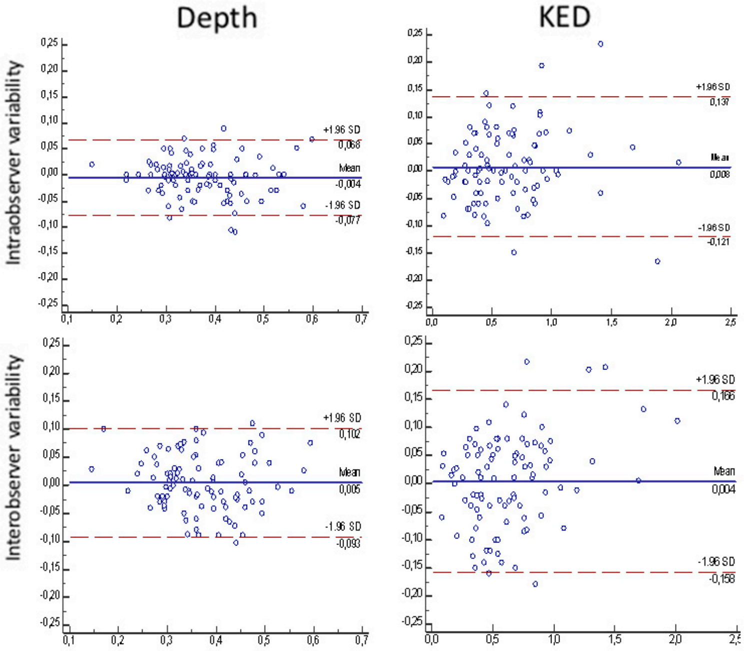

Figure S4. Bland-Altman analysis for vortex depth and kinetic energy dissipation (KED) on 94 unselected, consecutive patients examined at Center 2. Abscissa and ordinate measures as described in Supplemental Figure S1. SD: standard deviation.

Repeatibility in patients with atrial fibrillation.			
	**Center 1 (N=13)**	**Center 2 (N=11)**	**Center 1 vs. Center 2**
Vortex area	ICC=0.88 (0.64-0.96)<br>LOA=0.07, -0.07	ICC=0.84 (0.50-0.95)<br>LOA=0.06, -0.06	ΔICC=0.04 (4.5%)
Vortex lenght	ICC=0.86 (0.58-0.96)<br>LOA=0.16, -0.14	ICC=0.84 (0.59-0.92)<br>LOA=0.06, -0.06	ΔICC=0.02 (2.3%)
Vortex depth	ICC=0.97 (0.92-0.99)<br>LOA=0.04, -0.06	ICC=0.94 (0.79-0.98)<br>LOA=0.06, -0.08	ΔICC=0.03 (3%)
Vortex intensity	ICC=0.91 (0.72-0.97)<br>LOA=0.04, -0.07	ICC=0.89 (0.64-0.96)<br>LOA=0.07, -0.08	ΔICC=0.02 (2%)
KED	ICC=0.99 (0.97-0.99)<br>LOA=0.11, -0.07	ICC=0.99 (0.97-0.99)<br>LOA=0.13, -0.11	ΔICC=0

Table S1. Repeatability of vortex flow measures in patients with atrial fibrillation. ICC: intraclass correlation coefficient. LOA: limits of agreement. ICC is reported with 95% confidence interval (in parentheses). ΔICC is reported as absolute difference and percentage (obtained by indexing Δ by the Center 1 value). KED: kinetic energy dissipation.

Reproducibility in patients with atrial fibrillation.			
	**Center 1 (N=13)**	**Center 2 (N=11)**	**Center 1 vs. Center 2**
Vortex area	ICC=0.81 (0.46-0.94)<br>LOA=0.11, -0.07	ICC=0.82 (0.45-0.93)<br>LOA=0.07, -0.07	ΔICC=0.01 (1%)
Vortex lenght	ICC=0.91 (0.75-0.98)<br>LOA=0.13, -0.11	ICC=0.87 (0.60-0.96)<br>LOA=0.10, -0.07	ΔICC=0.04 (4%)
Vortex depth	ICC=0.95 (0.84-0.98)<br>LOA=0.09, -0.05	ICC=0.88 (0.85-0.93)<br>LOA=0.06, -0.07	ΔICC=0.07 (7%)
Vortex intensity	ICC=0.92 (0.76-0.98)<br>LOA=0.05, -0.05	ICC=0.91 (0.69-0.63)<br>LOA=0.06, -0.10	ΔICC=0.01 (1%)
KED	ICC=0.97 (0.91-0.99)<br>LOA=0.16, -0.17	ICC=0.99 (0.96-0.99)<br>LOA=0.15, -0.11	ΔICC=0.02 (2%)

Table S2. Reproducibility of vortex flow measures in patients with atrial fibrillation. ICC: intraclass correlation coefficient. LOA: limits of agreement. ICC is reported with 95% confidence interval (in parentheses). ΔICC is reported as absolute difference and percentage (obtained by indexing Δ by the Center 1 value). KED: kinetic energy dissipation.
